# Supplementary material for: Exploring women’s decisions of where to give birth in the Peruvian Amazon; why do women continue to give birth at home? A qualitative study
Source: PLoS One. 2021 Sep 10;16(9):e0257135. doi: 10.1371/journal.pone.0257135 (PMC8432815; doi:10.1371/journal.pone.0257135)
Supplement: S1 File — (PDF) [file pone.0257135.s001.pdf]

Consolidated criteria for reporting qualitative studies (COREQ): 32-item checklist

Developed from: Tong A, Sainsbury P, Craig J. Consolidated criteria for reporting qualitative research (COREQ): a 32-item checklist for interviews and focus groups. International Journal for Quality in Health Care. 2007. Volume 19, Number 6: pp. 349 – 357

Topic	Item No.	Guide Questions/Description	Reported on Page No. (and description)
**Domain 1: Research team and reflexivity**			
Personal characteristics			
Interviewer/facilitator	1	Which author/s conducted the interview or focus group?	9, 27 (Interviews conducted by lead researcher EG)
Credentials	2	What were the researcher's credentials? E.g. PhD, MD	27 – Medical student
Occupation	3	What was their occupation at the time of the study?	27 – Medical student
Gender	4	Was the researcher male or female?	27 – Female
Experience with training	5	What experience or training did the researcher have?	N/A
Relationship with participants			
Relationship established	6	Was a relationship established prior to study commencement?	47
Participant knowledge of the interviewer	7	What did the participants know about the researcher? E.g. personal goals, reasons for doing the research	47
Interviewer characteristics	8	What characteristics were reported about the interviewer/facilitator? E.g. Bias, assumptions, reasons and interests in the research topic	23
**Domain 2: Study Design**			
Theoretical Framework			
Methodological orientation and Theory	9	What methodological orientation was stated to underpin the study? E.g. grounded theory, discourse analysis, ethnography, phenomenology, content analysis	10
Participant selection			

Topic	Item No.	Guide Questions/descriptions	Reported on Page No. (and description)
Sampling	10	How were participants selected? E.g. purposive, convenience, consecutive, snowball	8 – convenience<br>A snowball recruitment strategy was also utilised, but no participants were enrolled through this method
Method of approach?	11	How were participants approached? E.g. face-to-face, telephone, mail, email	8
Sample size	12	How many participants were in the study?	2, 9 (n=25)
Non-participation	13	How many people refused to participate or dropped out? Reasons?	N/A
Setting			
Setting of data collection	14	Where was the data collected? E.g. home, clinic, workplace	8, 47
Presence of non-participants	15	Was anyone else present besides the participants and researchers?	9, 23, 47 (interpreter)
Description of sample	16	What are the important characteristics of the sample? E.g. demographic data, date	8
Data collection			
Interview guide	17	Were questions, prompts, guides provided by the authors? Was it pilot tested?	48 – Summary of topic guide provided – 1 pilot interview conducted. The topic guide was then developed iteratively
Repeat interviews	18	Were repeat interviews carried out? If yes, how many?	N/A
Audio/visual recording	19	Did the research use audio or visual recording to collect the data?	10
Field notes	20	Were field notes made during and/or after the interview or focus group?	10
Duration	21	What was the duration of the interview or focus group?	9
Data saturation	22	Was data saturation discussed?	10

Topic	Item No.	Guide Questions/descriptions	Reported on Page No. (and description)
Transcripts returned	23	Were transcripts returned to participants for comment and/or correction?	23
**Domain 3: analysis and findings**			
Data analysis			
Number of data coders	24	How many data coders coded the data?	10 – principal researcher + 2 research assistants dual coded a sample of transcripts
Description of the coding tree	25	Did authors provide a description of the coding tree?	11 – table of themes and subthemes provided
Derivation of themes	26	Were themes identified in advance or derived from the data?	10 – themes were derived from the data however coding was performed inductively and deductively
Software	27	What software, if applicable, was used to manage the data?	10 – NIVO 12
Participant checking	28	Did participants provide feedback on the findings?	N/A
Reporting			
Quotations presented	29	Were participant quotations presented to illustrate the themes/findings? Was each quotation identified? E.g. participant number	11-20
Data and findings consistent	30	Was there consistency between the data presented and the findings?	11-25
Clarity of major themes	31	Were major themes clearly presented in the findings?	11
Clarity of minor themes	32	Is there a description of diverse cases or discussion of minor themes?	11 (table) and 11-25
