# Supplementary figures and images for: Exploring women’s decisions of where to give birth in the Peruvian Amazon; why do women continue to give birth at home? A qualitative study
Source: PLoS One. 2021 Sep 10;16(9):e0257135. doi: 10.1371/journal.pone.0257135 (PMC8432815; doi:10.1371/journal.pone.0257135)

## Visualisation of the Recruitment Process

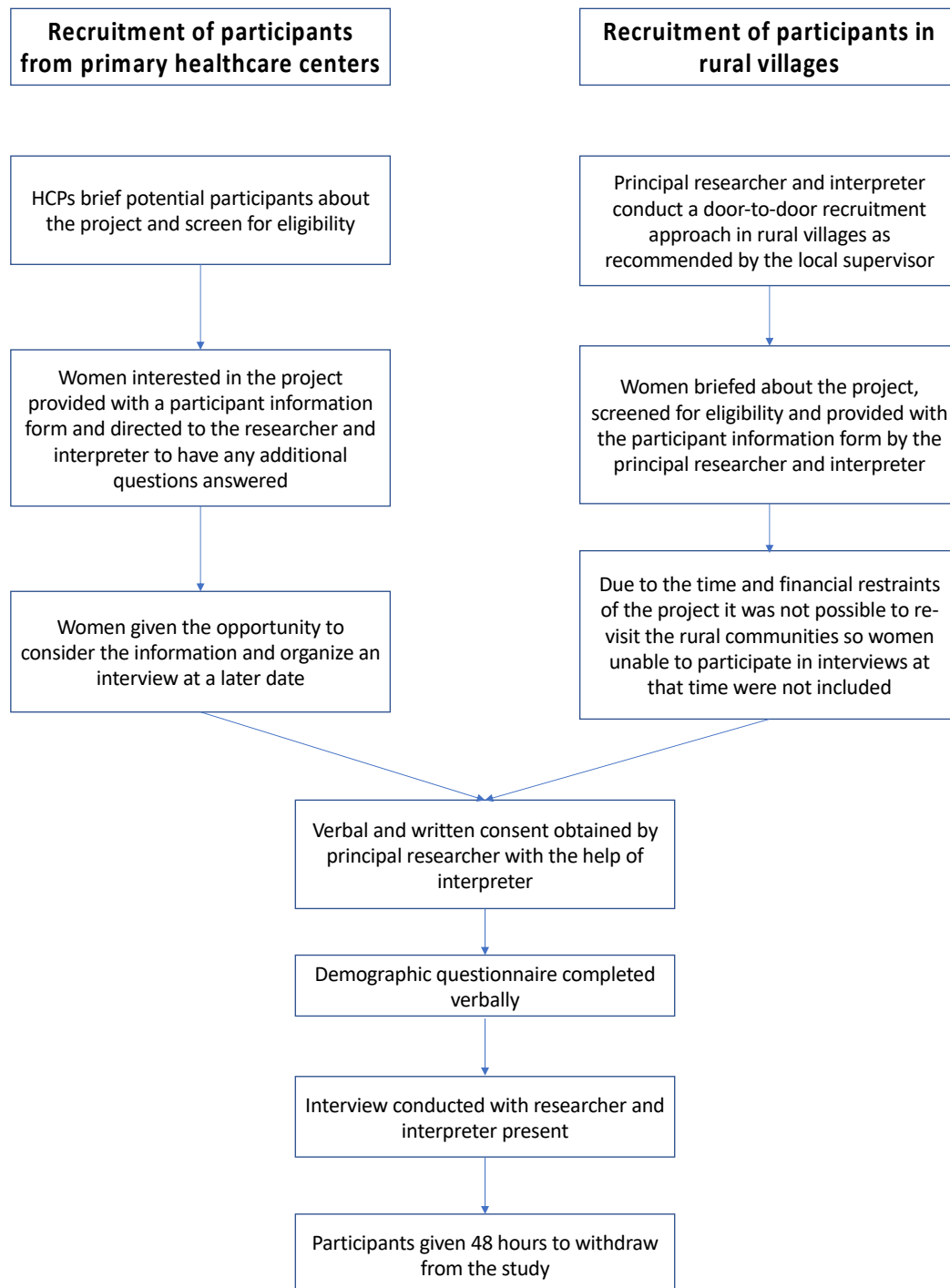

Supplement: S2 File — (PDF) [file pone.0257135.s002.pdf]
