# Supplementary material for: Exploring women’s decisions of where to give birth in the Peruvian Amazon; why do women continue to give birth at home? A qualitative study
Source: PLoS One. 2021 Sep 10;16(9):e0257135. doi: 10.1371/journal.pone.0257135 (PMC8432815; doi:10.1371/journal.pone.0257135)
Supplement: S3 File — (PDF) [file pone.0257135.s003.pdf]

Summary of topic guide

Preceding each interview

1. Introduce researcher and interpreter
2. Explain the purpose of this study
3. Explain the logistics of the interview including time, content and answer any questions
4. Obtain written consent
5. Complete the demographic questionnaire
6. Confirm permission to audio-record.

Topic	Questions and probes
**Locations of delivery**	Location of recent birth (e.g. home, centro de salud, hospital)<br>Decision process surrounding location<br>Other previous homebirth locations<br>Information/advice about possible locations for childbirth<br>ANC attendance during pregnancy<br>Why/why not?<br>Discussion of delivery locations
**Birth experience**	Day of childbirth. Location, aspects of care (positive and negative), care providers/birth partners.<br>Differences from prior births
**Phase 1 barriers**	Awareness of risks/diseases associated with pregnancy and childbirth<br>Prior delivery locations – influence<br>Prior healthcare exposure and experiences<br>Other beliefs influence – religion, culture<br>“Normal” place for childbirth
**Phase 2 barriers**	Access to transport – Influence on birth location<br>Normal methods of accessing healthcare
**Phase 3 barriers**	Highs and lows of IPC<br>Anything that would have been preferred?<br>General beliefs to do with hospitals/postas. e.g., interventions, stigma, help, pain relief
**Closing factors**	Are there any other factors which influenced your place of birth which we have not discussed?<br>Why do you believe home births are more common in Loreto than the rest of Peru?<br>If you were to have another child, where would you like to give birth?
**End**	Thank, questions, right to withdraw information, signpost, snowball recruitment
