# Supplementary material for: Exploring women’s decisions of where to give birth in the Peruvian Amazon; why do women continue to give birth at home? A qualitative study
Source: PLoS One. 2021 Sep 10;16(9):e0257135. doi: 10.1371/journal.pone.0257135 (PMC8432815; doi:10.1371/journal.pone.0257135)
Supplement: S4 File — (DOCX) [file pone.0257135.s004.docx]

**Themes, Subthemes & Codes Breakdown**

| **Theme** | **Subthemes** | **Codes** | **Notes** |
| --- | --- | --- | --- |
|  | | | |
| **Financial barriers** | Insurance | Provisions of insurance, lack of insurance, insufficiencies of insurance systems | *Women interviewed had access to both EsSalud and SIS, however gaps within the insurance existed including medications, transport to hospital. Women often reported unexpected costs including ‘sheets’ for their beds, fluids and medications required for the newborn.* |
|  | Additional costs | Medication costs, transport costs |  |
|  | Poverty | Unemployment, lack of costs with homebirths |  |
|  | | | |
| **Accessing care** | Transport | Poor availability, night time transport, transport costs, only public transport | *Access to care broadly falls under Thaddeus and Maine’s Phase 2 delays. It varied from the availability of transport (particularly at night and included ambulances) to the physical distance itself. The distance often gave rise to fear of delivering en route to healthcare facilities. Varying features of labour also influenced women’s ability to access care, including timing at night, the speed of labour (i.e. quick onset) as well as their ability to walk to reach transport.* |
|  | Distance | Distance by road, fear of delivering en route, travel to Iquitos prior to birth |  |
|  | Nature of labour – speed and pain of labour | Pain during labour, night time labour, speed of labour |  |
|  | | | |
| **Fear of healthcare facilities** | Fear of hospital | Unfamiliarity with hospital, prior experiences of healthcare, general fear of hospital | *Fear of healthcare many women’s decisions process. This included a general fear of the environment through to fear of episiotomies and caesarean sections. Additionally, experiences (both personal and of others) of poor and abusive care, including verbal abuse and excessive vaginal examinations.* |
|  | Fear of interventions | Fear of cutting, fear of caesarean, desire to have natural birth |  |
|  | Discomfort with hospital care | Embarrassment of exposure, abusive behaviour, fear of touching |  |
|  | | | |
| **Importance of seeking care** | Realisation of the importance of care | Fear of homebirth, fear from stories of homebirths, availability of medical equipment, safety of hospital | *There was mixed awareness about the importance of intrapartum care as well as complications during the antenatal period and birth. Some women demonstrated awareness of the availability of medical equipment in the healthcare setting. However many women, often those choosing to have a homebirth were not aware. Advice surrounding complications during pregnancy and place of delivery was mixed from HCPs. A selection of women also deemed that from ‘normal’ ANC appointments, they were therefore not at risk of complications during labour.* |
|  | Poor education | Awareness of diseases and illnesses, insufficient information |  |
|  | Healthcare Practitioner’s advice | Information from HCPs, absence of staff at antenatal care, expectance of a normal birth |  |
|  | | | |
| **Comfort and traditions of home** | Comfort of home | Cultural and traditional practices, normality of homebirth, positive homebirth experiences | *The comfort, traditions and normality of being at home for labour was an obvious reason for women to select to remain at home for delivery. This covered cultural traditions such as herbal remedies, the experience of family members including elders and the support available at home. As well as this the ease of staying at home for continuation of housework and the provision of care for other children. Furthermore, the comfort of home avoided hospital experiences and women reported greater autonomy including in birthing position.* |
|  | Care available at home | Family support, postnatal care following homebirth |  |
|  | Continuation of daily activities | Continuation of jobs, care for other children |  |
|  | Avoiding hospital experiences | Inability to breastfeed, general fear of hospital, fear of cutting, fear of caesarean |  |
|  | Autonomy | Choice of birthing position, fear of caesarean |  |
